# Ion release, biocompatibility, and bioactivity of resin-modified calcium hydroxide cavity liners

**DOI:** 10.1186/s12903-023-03723-3

**Published:** 2023-12-21

**Authors:** Nastaran Taghvaei, Mehrsima Ghavami-Lahiji, Mehdi Evazalipour, Reza Tayefeh Davalloo, Ehsan Zamani

**Affiliations:** 1 Dental Sciences Research Center, School of Dentistry, Guilan University of Medical Sciences, Rasht, Iran; 2 Department of Restorative Dentistry, Dental Sciences Research Center, School of Dentistry, Guilan University of Medical Sciences, Rasht, Iran; 3 Department of Pharmaceutical Biotechnology, School of Pharmacy, Guilan University of Medical Sciences, Rasht, Iran; 4 Department of Pharmacology and Toxicology, School of Pharmacy, Guilan University of Medical Sciences, Rasht, Iran

**Keywords:** Calcium hydroxide, Dental Cavity Liner, Biocompatibility Testing, Calcium release, Solubility

## Abstract

**Background:**

The placement of liners near the pulp area is essential for therapeutic effects and maintaining pulp health while stimulating the formation of tertiary dentin. This in vitro study aimed to evaluate the calcium release, pH, biocompatibility, solubility, and bioactivity of three resin-modified calcium hydroxide cavity liners.

**Methods:**

The disc specimens of each cavity liner were prepared using polyethylene molds of 7 mm in diameter and 2 mm in height (*n* = 10). Three light-cure liners evaluated include Ultra-Blend Plus (UB), Base-it (BI), and Master Dent (MD). The samples were then immersed in flasks containing 10 mL of distilled water. Calcium ion release, pH, and solubility were evaluated in two weeks of incubation. The cytotoxicity of extracts adjacent to the specimens was evaluated by MTT assay using NIH/3T3 cells after 1, 3, and 7 days of incubation. The ability to induce the nucleation of calcium phosphates (CaPs) after 28-day immersion in a simulated body fluid was investigated by SEM-EDX analysis. Statistical analysis was performed using ANOVA, Kruskal-Wallis, and repeated measures tests at the significant level of 0.05.

**Results:**

There was a significant difference in the release of calcium ions among the three liners investigated on days 1, 7, and 14 (*p* < 0.05). UB liners exhibited a significantly higher amount of calcium release than the other two liners, followed by BI, and MD. On day 1, there was no significant difference in the average pH among the three liners. However, after day 7, the MD liner showed a significant decrease in pH compared to the other two liners. BI liner demonstrated the highest level of biocompatibility, followed by the MD and UB liners. UB showed a high calcium release, solubility with no alkalizing activity, and the formation of more mature Ca-rich apatite deposits than the other two liners.

**Conclusion:**

Based on the results of this study, the cavity liner material’s performance is material dependent. It can impact ion release, biocompatibility, and bioactivity which are important factors to consider in clinical practice. Further studies are needed to investigate the long-term effects of different liner materials on oral tissues.

## Introduction

The preservation of pulp vitality in compromised teeth is one of the goals of operative dentistry; one technique used for this purpose is the use of liners and bases. This entails applying protective materials to the area near the pulp to preserve its health and encourage tertiary dentine deposition’s defensive repair process. Since many years ago, it has been a standard procedure to use liners and bases underneath restorations in deep cavities, and operative dentistry textbooks still emphasize this use as a crucial component of restorative practices [1].

As our understanding of teeth and dental materials grows, the ideas surrounding pulp protection are continually being explored. Liners have historically been used to protect the pulp from the potentially toxic effects of restorative substances. Liners are currently employed for their therapeutic effects as well as to seal the dentinal tubules against the entrance of microorganisms or their byproducts at the tooth-restoration interface [1, 2].

Today, the use of liners in deep cavities and cap pulp treatments is considered an inevitable part of conservative dentistry.

To protect pulp tissue from irritations caused by the restorative procedure, a variety of dental materials have been introduced as liners [1, 3]. Calcium hydroxide, glass ionomers, and resin-modified glass ionomers (RMGIs) are examples of common lining materials. For a long time, calcium hydroxide has been regarded as the gold standard and is the most widely used by general dentists. Calcium hydroxide has reportedly been the preferred liner when treating patients with deep cavities [4–6].

However, conventional calcium hydroxide liners suffer from significant drawbacks such as low elastic modulus, low compressive strength, and high solubility [1].

The modifications made to the new generation of calcium hydroxide-based materials attempt to do away with calcium hydroxide’s drawbacks and improve its physicochemical characteristics [3].

Owing to the limited mechanical properties of chemically cured calcium hydroxide and the need to use this material in areas near the pulp, light-curable calcium hydroxide-based cements made of methacrylate monomers have been developed. These cements have the following benefits: controlled working time, increased resistance, low solubility in acid, and immediate attainment of optimal mechanical properties after curing [7].

However, when resin-incorporated materials are used in deep cavities, unpolymerized monomers may diffuse into the pulp tissues through the dentinal tubules [8–11]. If the monomers come into direct contact with the dental pulp, the situation could get worse [12]. Thus, to maximize the physical properties and clinical effectiveness of light-curable calcium hydroxide cavity liners, a long-lasting bacterial seal, the release of essential ions to promote pulp tissue healing, and mineralized tissue formation without imposing toxic effects are necessary [7].

Improvements have also been made in calcium silicate-based materials with excellent biocompatibility properties. For example, TheraCal LC is a resin-modified calcium silicate-based material designed for direct and indirect pulp protective materials under dental restorations [13, 14].

It is worth noting that the addition of light-curable monomers gives the material the ability to be command-cured and prevents issues with the bonding of composite resin to the underlying liner [7, 15].

Different studies have confirmed the appropriate biocompatibility of various types of calcium silicates; however, there is no comprehensive information regarding resin-modified calcium hydroxide cavity liners. Additionally, inconsistent findings on the calcium release rate and biocompatibility of resin-modified calcium hydroxide liners have been reported in the literature that is currently available [16, 17].

So, the lack of sufficient information related to the issues raised and the need for a more comprehensive search regarding this type of liner that is widely used in restorative dentistry due to its greater variety and lower price in the market compared to that of the calcium silicate family, led to the design of the present study. It is necessary to evaluate the Ca release potential of these materials. However, ion release in combination with cytocompatibility has not been studied in resin-modified calcium hydroxide liners yet.

This study aimed to assess the properties of resin-modified calcium hydroxide cavity liners, including Ultra-Blend Plus (UB), Base-it (BI), and Master Dent (MD), by using the following five outcome measures: (1) the amount of calcium released from these capping materials; (2) the pH values of aqueous medium exposed to the extracts of the capping materials that stimulate pulp cell differentiation; (3) the viability of the cells as determined by an MTT assay; (4) solubility, and (5) bioactivity of these capping materials.

## Materials and methods

### Measurement of calcium ion release

The disc specimens of each resin-modified calcium hydroxide cavity liner were prepared using polyethylene molds (*n* = 10). The molds with dimensions of 7 mm in diameter and 2 mm in height were filled with the cavity liner (Table 1). Both sides of the mold were covered by Mylar sheets to prevent an oxygen-inhibited layer.

**Table 1:** Resin-modified Calcium hydroxide-based cavity liners used in this study

	Composition	Lot	Manufacturer
Base-it (BI)	calcium hydroxyapatite in urethane methacrylate oligomer	BI20003	Spident
Ultra-Blend Plus (UB)	Calcium hydroxide and calcium hydroxyapatite in a urethane dimethacrylate base	BJLK8	Ultradent
Master Dent (MD)	Hydroxyapatite, barium sulfate and fluoride	12,068	Dentonics

The specimens were cured with a light-curing unit (LED.F, Woodpecker, China) (1200 mW/cm^2^) for 20 s on top and bottom surfaces, as recommended by the manufacturer. The light intensity of the light-curing unit was checked using a radiometer (Woodpecker, Medical Instrument, China).

The specimens were immersed in the flasks containing 10 mL of distilled water at 37 °C at a relative humidity of 100% for 1, 7, and 14 days [17]. The amount of calcium ion released from the cavity liners in the distilled water was measured by inductively coupled plasma atomic emission spectroscopy (ICP OES 730-ES; Varian, USA) [13].

After each period, the specimens were removed and transferred to new flasks with 10 mL of distilled water. The solutions contained in the flask were used after each experimental period.

The standard code for calcium measurement tests is inorganic ventures CGCA 10 − 1. Before the test, HNO_3_ was added to the samples to reach a concentration of 1%. The device was calibrated at 0.1, 0.5, 1, 5, 10, 50, 100, and 200 ppm calcium concentrations. By absorbing energy, the electrons around the nucleus of the atom go to higher energy levels, and the atom is in an excited state. In this device, the excitation source is inductively coupled plasma (ICP). The energy of this type of induced plasma creates a variable electromagnetic field. After some time, due to the instability of the excited atom, the electron moves to lower levels, and the energy difference between the two energy levels is emitted as electromagnetic radiation with a specific energy and wavelength [18].

Examining the energy of this radiation can reveal information regarding the type of atoms involved in this process. For calcium atoms, this energy is released in the form of electromagnetic radiation with a wavelength of 396.847 nm, and the calcium concentration is measured by the device based on the intensity of the emitted wavelength [18].

### Measurement of pH

Specimens were prepared as described for the Ca release test. The specimens were then immersed in flasks containing 10 mL distilled water at 37 °C and relative humidity 100% for 1, 7, and 14 days. The pH values were evaluated by a digital pHmeter (827 pH/ion meter, Metrohm, Switzerland), which was previously calibrated with buffer solutions (pH 4.0 and 7.0) [13].

### Measurement of solubility

The specimens were prepared as mentioned above. The specimens were subjected to a solubility test. Each specimen’s initial dry weight (W0) was determined using an analytical lab balance (Wisd WBA-320, Witeg, Germany), after which it was submerged for 14 days at 37 °C in a vial containing 10 mL of distilled water. The specimens were taken out of the media, dried with filter paper, held in a vacuum desiccator, and reweighed (W1). The differences found between these two weights were divided by the initial dry weight of the samples, multiplied by 100, and reported as a solubility percentage [19, 20].

### Cell culture

The cells used in this study were NIH/3T3 mouse fibroblasts (NCBI No. *C156*; *Pasture* Institute, Iran) cultured in Dulbecco’s Modified Eagle Media (DMEM; Ideh zistnotarkib, Iran) [7, 15]. This medium was supplemented with 10% fetal bovine serum (FBS 10%, GibcoTM, England), penicillin, and streptomycin and incubated for 24 h at 37 °C with 5% CO_2_ (Incubator, Memmert, Germany).

Cells were initially passaged on culture flasks by inducing fibroblast proliferation and changing the culture medium. Once ~ 80% confluency has been reached and cells adhere to the flask, trypsin/ethylenediamine tetraacetic acid (EDTA) solution (Ideh zistnotarkib, Iran) was applied for 2 min at 37 °C to detach the cells.

Culture medium was added to the live cells and centrifuged to form a pellet. After adding new culture medium and pipetage, 100 µl of pellet containing 10,000 3T3 cells were seeded in 96-well plates (Cell Culture Microplate 96-well, SPL, South Korea) and incubated for 24 h at 37 °C and 5% CO_2_.

### Biocompatibility test

Specimens were prepared as mentioned for the Ca release test and then sterilized at a laminar flow hood (Behrad Sanat, Iran) for 20 min under UV light. To investigate the toxicity of the cavity liners and their effect on cell growth and proliferation, the extraction process was performed according to ISO 10993-12 (Tests for In Vitro Cytotoxicity). Immediately after the mixing and curing process, the samples of the three groups were immersed in 400 µl of DMEM culture medium, whereby the ratio of sample surface area to medium was 3 cm^2^/mL [21].

The extracts obtained from the samples were tested for cell viability after 24 h, 3 days, and 7 days of remaining in the incubator. The extracts from this stage were stored, and 100 µl of them were transferred to each well in a 96-well culture plate containing cells (as mentioned in the Cell culture section).

Culture medium (DMEM) containing cells without the extract of specimens was also considered as a control. After incubation for 48 h, the medium was aspirated, and 100 µl of MTT solution was added to the medium.

Then, culture medium was removed, and 100 µl of MTT at a concentration of 0.5 mg/ml was poured into each well and incubated for 3 h. Culture medium containing MTT was taken out of the wells, and 150 µl of dimethyl sulfoxide (DMSO) was added to dissolve its formazan crystals. Finally, the absorbance of the MTT solution was read using an ELISA Microplate Reader (ELISA reader, Epoch, Germany) at 570 nm and compared with control values. Cell viability is calculated via the following equation:

$$Cell\;Viability\;\left(\%\right)=\frac{{Mean\;OD}_{sample}}{{Mean\;OD}_{control}}\times100$$

Data were analyzed by one-way ANOVA, Kruskal-Wallis, repeated measures tests, and Bonferroni correction (α = 0.05).

### 5. EDX surface analysis

Two specimens of each liner were immediately immersed vertically in 20 mL of simulated body fluid (SBF) and stored at 37 °C for 7 and 28 days. The medium was renewed weekly with fresh SBF. The specimens were examined using scanning electron microscopy-energy-dispersive X-ray (SEM/EDX) analysis (TESCAN, VEGA2, Czech Republic) after 7 and 28 days to evaluate the impact of long-term immersion on the mineral content formed on the surface [22, 23].

The Ca/P atomic ratio taken from the surface of the specimens was calculated and compared to the Ca/P atomic ratio in apatitic and nonapatitic CaPs, including Ca-poor apatitic (Ca/P 1.5–1.67), Ca-poor nonapatitic CaPs (Ca/P ratio < 1.47), calcium-rich (carbonated) CaPs (Ca/P ratio 1.6–2.0), and calcium-rich non-apatite CaPs (Ca/P 1.83).When Ca/P ratios are very high, the formation of calcium carbonate isomorphs such as calcite and aragonite is considered [23, 24].

## Results

**Fig. 1:**
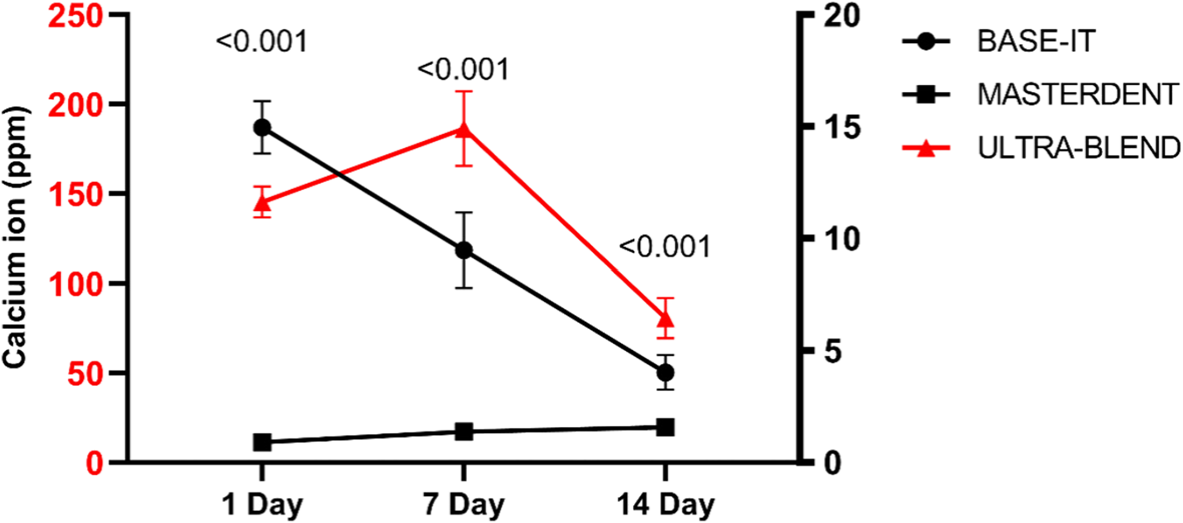
EDX analysis of liners after 7 and 28 days of immersion in SBF

Tables 2 and 3 show the calcium release, PH values, and Solubility obtained for three calcium hydroxide-based liners, respectively. The amount of calcium released on the first day by the Ultra-Blend Plus (UB) liner was significantly higher than that of the two other liners (145.38 ± 8.69 ppm) (*p* < 0.05). The calcium release from UB increased for up to 7 days and thereafter decreased to 80.68 ± 11.10 ppm. MD revealed the lowest calcium release, with a statistically significant difference between UB and BI (*p* < 0.05) and remained almost unchanged throughout the experiment. Moreover, BI followed a decreasing trend during the experiment, from 14.96 ± 1.17 ppm to 4.03 ± 0.77 ppm. There were significant differences in calcium release among UB, BI, and MD (Table 2) (Fig. 1). Solubility after 14 days by the UB liner was significantly higher than that of the two other liners (*p* < 0.05) (Table 1).

**Table 2:** Calcium release and solubility of materials for the time-points tested (ppm) (Mean ± SD).

Cavity liner	Calcium release			Solubility
	24 h	7 days	14 days	14 days
Base-it (BI)	14.96 ± 1.17	9.47 ± 1.68	4.03 ± 0.77	1.21 ± 0.51
Ultra-Blend Plus (UB)	145.38 ± 8 69	186.31 ± 20.85	80.68 ± 11.10	5.21 ± 0.90
Master Dent (MD)	0.90 ± 0.09	1.37 ± 0.06	1.58 ± 0.10	1.52 ± 0.57

**Table 3:** pH values of leachates of liners for the time-points tested (Mean ± SD).

Cavity liner	Time points		
	24 h	7 days	14 days
Base-it (BI)	6.49 ± 0.08	6.39 ± 0.04	6.28 ± 0.06
Ultra-Blend Plus (UB)	6.57 ± 0.06	6.45 ± 0.03	6.43 ± 0.03
Master Dent (MD)	6.34 ± 0.42	6.14 ± 0.20	6.07 ± 0.12

**Fig. 2:**
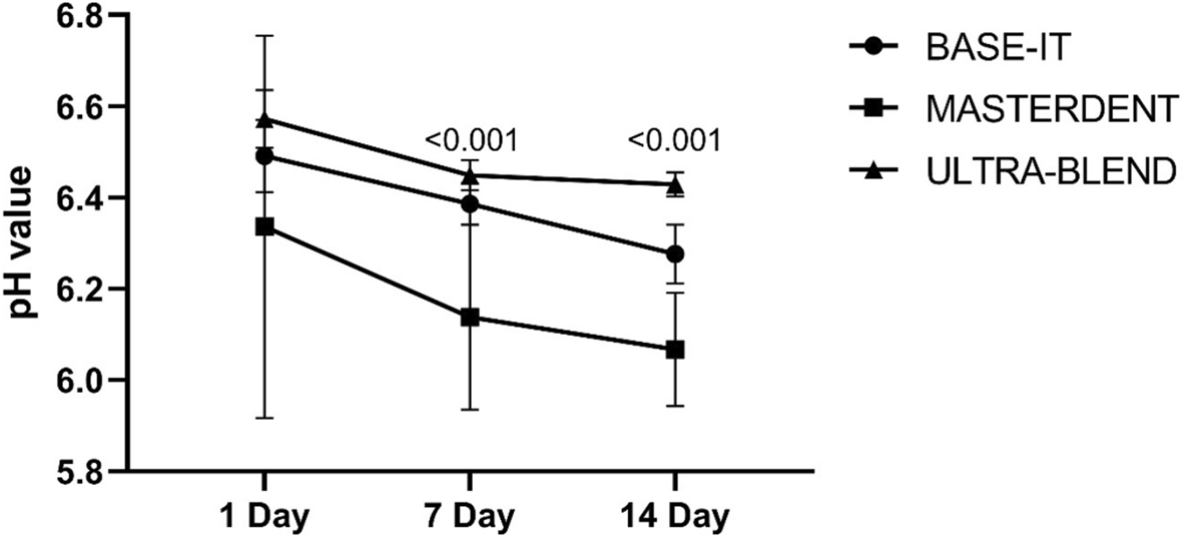
pH values of leachates of liners for the time points tested

There was no significant difference in the average pH among the three liners examined on day 1; however, after day 7, a significant decrease was observed in the case of MD in comparison with the two other liners (Table 3). On the 14 days, there were significant statistical differences between MD and BI (*p* = 0.038), between UB and MD (*p* < 0.001), and between UB and BI (*p* < 0.031). The average pH in MD was lower than the other two groups, and the average pH in BI was lower than UB (Fig. 2).

**Fig. 3:**
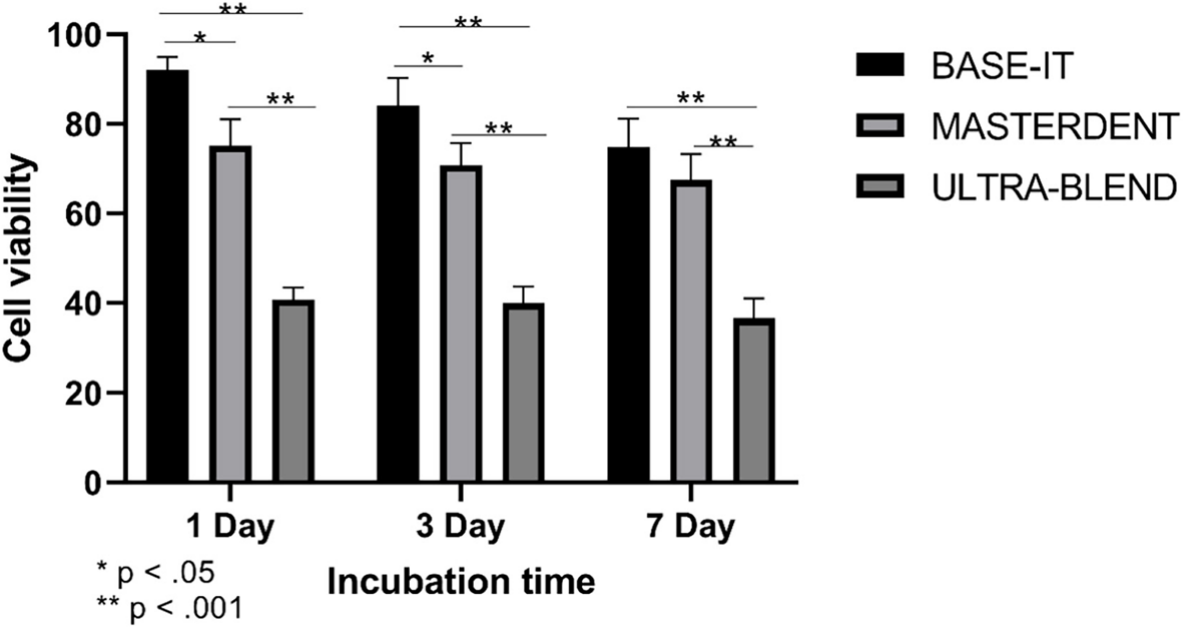
Cell viability of leachates of liners for the time points tested

Figure 3 depicts the cell viability results obtained for the three-calcium hydroxide-based cavity liners. BI showed the highest cell viability compared to that of UB and MD in all three time periods tested. UB showed significantly lower cell viability than others (*p* < 0.05). MD presented lower cell viability compared to that of BI and a higher viability rate than UB. All pairwise comparisons yielded significant differences; however, there was no significant difference between BI and MD at 7 days (*p* > 0.05). All tested materials showed a decreasing trend of cell viability with increasing immersion time in the culture medium.

**Fig. 4:**
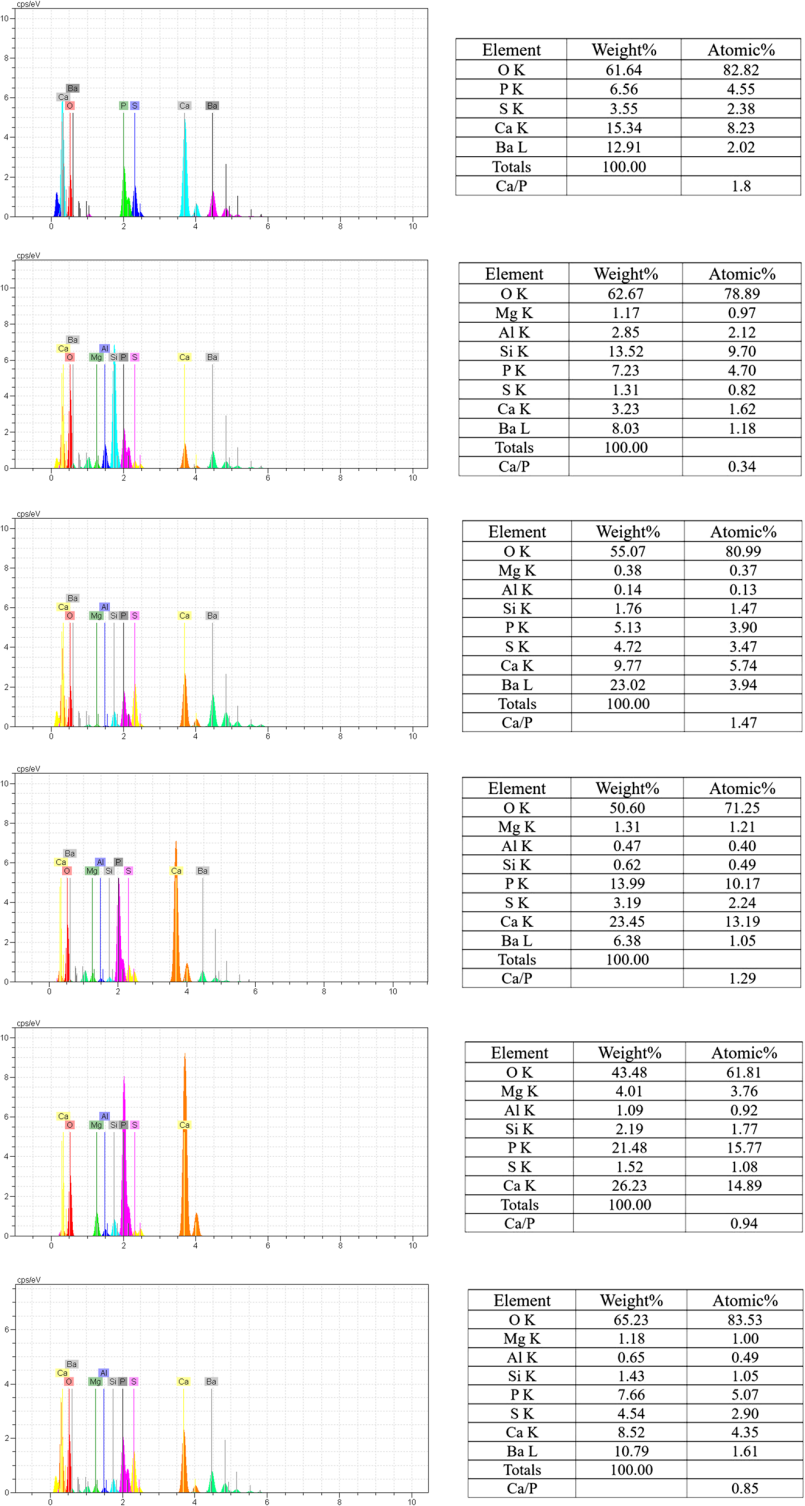
EDX analysis of liners after 7 and 28 days of immersion in SBF

In our study, UB showed a high Ca release (23.45% wt) after 28 days, correlated with the presence of a higher amount on the surfaces than the other two materials. EDX analyses disclosed Ca, silicon (Si), phosphorus (P), aluminum (Al), magnesium (Mg), sulfur (S), oxygen (O), and barium (Ba). The radiopacifier barium sulfate that existed in their formulation may explain the observed Ba (6.38% wt) and S (3.19% wt), and the silica-containing urethane may explain Si (0.62% wt) components in EDX analysis [22] (Fig. 4).

The Ca/P atomic ratio for BI was approximately 0.34 at 7 days and slightly increased to approximately 0.94 after 28 days in SBF, suggesting that at any time the deposit was constituted by nonapatitic Ca-poor CaPs. The Ca/P atomic ratio for MD decreased with the soaking time from approximately 1.47 at 7 days and finally to 0.85 after 28 days in SBF, suggesting that the deposit was composed of nonapatitic Ca-poor CaPs [23]. MD and BI had low calcium release and less CaPs deposition after aging in SBF.

On the contrary, UB showed a high calcium release with no alkalizing activity, and the formation of more mature Ca-rich apatitic precursors was correlated with their higher ion-releasing ability (Ca/P atomic ratio = 1.8). However, it decreased after 7 days, which corresponds well with the decreasing amount of Ca released after 7 days.

## Discussion

The resin-modified calcium hydroxide system has several advantages over conventional one-paste or two-paste calcium hydroxide systems, including superior physical properties, light polymerization, less affected by phosphoric acid, and low water solubility over the course of time. It has been reported that unpolymerized resins and monomers may be toxic to pulp cells [1, 3]. The number of studies that have examined the biocompatibility factors, calcium release, and hydroxyl ions in resin-modified calcium hydroxide liners is also limited. Most of the studies that investigated the characteristics above are only on the UB brand [7, 17] or on materials with calcium-silicate base [13, 15].

And there is no comprehensive information about different resin-modified calcium hydroxide cavity liners in the market. Because today the use of these liners as conservative and easy treatment is favored by dentists and considering that they are easy to use and have a more reasonable price than calcium silicate-based materials, in this study, three light-cure liners with calcium base Hydroxide available were investigated.

Cavity liners based on calcium hydroxide release calcium and hydroxyl ions, which have antibacterial effects and promote mineralization, respectively [3, 15]. Calcium release has been linked to the biological characteristics of calcium hydroxide cement and hydraulic cement because it promotes the differentiation potential of dental pulp cells and makes mineralization easier, which, over time, results in the deposition of a dentine-like barrier on the surface of the pulp [25]. In contrast, a prolonged leaching period may cause the material to have high solubility values, which may hinder the capacity of the restoration to seal. However, low solubility values may affect ion release [26–28].

Calcium hydroxide cement, proposed in 1930 as a remineralizing agent, stimulates pulp-derived cells and stem cells through the release of calcium and OH ions. This release increases pyrophosphatase activity, maintains dentin mineralization, and modulates osteopontin and BMP-2 levels during pulp calcification, which underlies human dental pulp cell proliferation and differentiation [16, 29].

Calcium ions significantly influence the biological processes involved in the neoformation of mineralized hard dental tissues. They activate adenosine triphosphate, increase bone-associated protein production, and promote dental pulp cell growth [30, 31].

Calcium neutralizes lactic acid generated by osteoclasts, inhibits endotoxin, and caused a superficial coagulation due to blood vessel damage [32]. Coagulation necrosis refines the matrix and aids odontoblast development. The permeability of the developing capillaries to calcium is similarly decreased by Ca ions. Because blood is the primary source of calcium for the formation of reparative dentin and calcium from Ca(OH)_2_ cement serves only as a stimulating agent, more calcium ions are retained during healing [16].

In the study of Natale et al. [33], it was reported that the rate of calcium release by calcium silicates is the highest, followed by self-cure calcium hydroxide cement. Pereira et al. [17], investigated the calcium release of light cure liners with calcium hydroxide bases such as UB and Biocal and a conventional calcium hydroxide liner, Hydro C. The highest amount of calcium release was seen in Hydro C. UB showed a lower amount of calcium release on the first day compared to Hydro C, but a similar release in 7 days. However, Biocal showed negligible calcium release. This means that the release of calcium in the conventional calcium hydroxide material was generally greater than that of the resin-modified type, and among the resin-modified liners, UB released more calcium ions than its other counterparts. The results of this study were consistent with our study that the UB Liner releases more calcium than other resin-modified liners with a calcium hydroxide base.

However, Chaudhari et al. [16] in a study measured the release rate of calcium ions in light-cured cement such as Hydrocal, Septocal, TheraCal, and Cal LC and compared them with self-cured calcium hydroxide cement, Dycal. The results of this study showed that Hydrocal and TheraCal cement released the highest amount of calcium. And the release of calcium ions by light-cured calcium hydroxide cement was calculated more than by self-cured calcium hydroxide cement, which contradicted the results of Pereira et al. [17] and our study.

It has been shown that hydraulic cement with tricalcium silicate bases like MTA and biodentine forms calcium silicate hydrate (CSH) and calcium hydroxide (CH) in contact with water. Generally, calcium ions leached from hydraulic cement derive mainly from the dissolution of the calcium hydroxide by-product and are higher than those in CH-based cement [13, 15]. Furthermore, the resin-modified form of these materials leaches calcium lower than their commercial resin-free counterparts [13, 15].

It has been reported that the release of hydroxyl ions also causes the release of alkaline phosphatase, which takes part in the mineralization process. Consequently, applying calcium hydroxide to the pulp tissue encourages repair and the emergence of a dentin bridge [17, 34]. The release of hydroxyl ions results in a high pH and promotes the enzymatic inhibition of microorganisms [16].

By causing an early necrotic layer, hydroxyl ions can stimulate pulp tissue healing. As a result, the relationship between the leaching profile and biological performance was examined, as well as the impact of various chemicals on calcium hydroxide release. Since bacteria can persist in the dentinal tubules after pulpal infections, pulp protection materials should have antimicrobial properties [35]. Because high alkalinity has bactericidal effects, continuous alkalinization is preferred [15].

Pereira et al. [17] in the study on light-cure liners with calcium hydroxide bases reported that the pH created by Hydro C > UB > Biocal. During the experiment, the pH of the material Hydro C was 10.59–10.76 in this study. In the UB material, the pH level was in the neutral range of 7.20–7.35, which was in line with the results of our study on all three resin-modified CH-based liner materials. Although the pH of Biocal material in the above study was reported in the acidic range of 5.25–5.57.

The majority of studies have confirmed an alkaline pH level for calcium silicate hydraulic cementitious materials (the MTA family) [13, 15], but there are few studies on materials with a calcium hydroxide resin-modified base, and there are material-dependent inconsistencies in the available studies [17, 20].

In Koutroulis et al.‘s study [15], the pH of each sample of commercial calcium silicate and experimental calcium silicate samples was checked after 24 h and on 7, 14, 21, and 28 days. ACTIVA BioACTIVE, which is a bioactive composite with ion-releasing properties, revealed that the lowest pH was approximately 8 among other specimens, followed by Theracal LC at approximately 12, and the rest of the samples had an almost equal and higher pH of approximately 14. All of these materials had higher pH values than those of our study materials.

In the present study, we employed fibroblast 3T3 for the biocompatibility test, according to Klein-Junior et al. [7] and Koutroulis et al. [15] In the study conducted by Klein-Junior et al. [7], they investigated the effect of previous heat treatment using a warm and hot air stream before light curing on the cytotoxicity of three light-cured calcium hydroxide-based cements and a conventional counterpart.

In the aforementioned study [7], the biocompatibility of liner UB was evaluated immediately after sample fabrication on the fibroblast cell, which was reported to be 10% on day 1 and 7% on day 7 at 37 °C, which was less than that of the other tested liners. Their findings were in line with our study, in which this material presented lower biocompatibility than the other two liners and was around 36–40%. The difference between the cell viability value and the present findings may be due to the study design, details of specimen fabrication, dimensions of the specimens, measurement methods, and immersion technique.

Some researchers have attributed the cytotoxicity caused by light-cured calcium hydroxide-based cement and dental adhesives possibly to residual resin monomers. With this type of dimethacrylate base material, the degree of conversion (DC) is about 70%. It has been predicted that about 9% of monomers may leach when in contact with fluids, considering the proportion of monomers that are not entirely converted and their potential diffusion into pulp tissues [3]. It is important to note that the conversion rate of dimethacrylate monomers to polymers of 70% does not correspond to 30% free monomers. It indicates that 30% of the methacrylate groups are still open to polymerization, but most of that 30% are now embedded in the polymer matrix. Only about 9% of monomers are free monomers, which can leach out because they include two uncured methacrylate groups in one monomer [3].

Contrary to our findings and Klein-Junior et al. [7], in vitro studies by Thunyakitpisal et al. [36] and Hirschman et al. [37] indicated that UB had no increase in cytotoxicity levels compared to the negative control.

The reason for the difference between the results of this study and previous studies on these substances, in which cytotoxicity was observed, is the incubation time before the start of the biocompatibility test [36, 37]. In the aforementioned studies, the materials were maintained at 37 °C for 72 h and 1 week, respectively, until the setting reaction was complete, after which a biocompatibility test was performed [36, 37]. Therefore, it seems that incomplete polymerization of materials and the presence of uncured components in studies that did not consider much time for post-curing reactions have a negative effect on the biocompatibility of a material.

The approach of different studies is diverse in this regard, and in some studies, they were placed in the media immediately or 4 h after the polymerization of pulp-capping materials. For example, Klein-Junior et al. immersed them in a DMEM solution immediately after preparing specimens [7]. This may be one of the reasons for the lower cell viability observed in this study than in other studies.

Kang’s study [13] on the biocompatibility of pulp capping materials was also performed 24 h after setting. Considering that during the treatment in clinical conditions, immediately after setting, the pulp capping materials were located near the dental pulp, we also considered the same conditions for the biocompatibility test, and the materials were examined immediately after setting.

Cytotoxic responses have been classified in the literature as severe (30%), moderate (30–60%), mild (60–90%), and nontoxic (> 90%) [38, 39]. According to Koutroulis’ research [15], calcium ion release has a moderately non-significant link with cytotoxicity but has a beneficial impact on antibacterial efficacy. Despite the maximum calcium release in our testing, the biocompatibility of UB dropped below that of the other materials. The BI liner showed the highest biocompatibility in the current investigation, and its results revealed that it had mild cell toxicity (~ 69–95%), with MD in second place (61–79%), and a negligible amount of calcium release, whereas moderate cytotoxicity was observed in the UB liner (32–43%). Although it has been claimed that the MTT assay cannot accurately simulate in vivo conditions, a higher concentration of materials can be tolerated because of the impact of a dynamic environment and the presence of buffering agents in the human body [40].

In addition, it is unlikely that the liner material placed on the tooth near the pulp will have dimensions similar to those of the samples prepared in this study and similar studies.

On the other hand, the fluid inside the dentinal tubules and the pulp tissue plays an important role in removing the toxins from these substances in the body by buffering. On average, the flow rate of dentinal tubules in the inward and outward states is about 849.9 μm/s to 460.4 μm/s [41], which can justify the positive clinical performance of this substance so far.

That is why, in some studies, extracts are diluted to different ratios to simulate in vivo conditions. For example, in the study by Koutroulis et al., the undiluted state of the extract, as well as 1/2, 1/4, and 1/8 dilutions, were used to evaluate the biocompatibility of paste coating materials [15].

Also, the cumulative amount of calcium released by the UB liner in 10 ml of distilled water on the tested days was approximately 421 ppm, while in the biocompatibility test, the culture medium adjacent to this liner was 400 µl. As stated by Maeno et al. [42], if the concentration of released calcium is higher than 10 mmol/L (400 ppm), it may have a cytotoxic effect, and it is possible that the concentration of calcium released in the culture medium was higher than 10 mmol/L. According to the literature, osteoblast stimulation occurs at a concentration of Ca ions of 2–4 mmol/L (80–160 ppm), and differentiation occurs at a concentration of 6–8 mmol/L [16, 42].

According to the results of this study, the Ca/P atomic ratio in BI and MD at 7 and 28 days were below < 1.47, consistent with non-apatitic CaP deposits, which demonstrated a fair capability to create CaP deposits (not properly defined as bioactivity), likely due to the inclusion of a methacrylate resin. However, UB showed the formation of more mature Ca-rich apatitic precursors correlated with their higher calcium-releasing ability and solubility (Ca/P atomic ratio = 1.8) [23, 24]. In addition, the pH level in the three liners was in the range of 6–7, but the release rate of calcium and solubility in UB was much higher than the others, which can be considered one of the possible reasons for the decrease in biocompatibility in this material. However, the degree of conversion was not measured in this study, and there is no information regarding unreacted monomers, which can be considered another possible cause of toxicity. It is said that if a layer of surface necrosis is created by the pulp capping material, neutrophils will infiltrate that area, and within a few weeks to a few months, due to the bioactivity of these materials, dystrophic calcification will cause the formation of tertiary dentin [43].

It must be emphasized that this finding should be interpreted cautiously because it does not necessarily reflect the in vivo situation on dental pulp stem cells. Furthermore, the information about resin-modified cavity liners from different companies is limited. So, there is still a need to study them according to the material-dependent nature of their properties. More research is being done to determine the degree of conversion and biologic evaluation in a setting that is closer to the real environment.

## Conclusions

Based on the results of the present study, it can be concluded that:

- The amount of calcium released by the UB liner was significantly higher, followed by the BI liner, and the lowest amount of calcium was released by the MD liner.
- The pH produced by the 3 test liners was almost identical and was in the neutral range of 5.6.
- The solubility by the UB liner was significantly higher, followed by MD, and BI.
- UB revealed deposition of more mature CaP apatitic precursors correlated with their higher ion releasing ability and solubility compared to that of MD and BI liners.
- BI liner biocompatibility was higher than others, followed by MD liner, which presented a mild range of toxicity. The biocompatibility of UB was significantly lower than the others and in the moderate toxicity range. However, these findings were obtained in laboratory conditions and cannot represent real clinical conditions.

## Data Availability

The datasets used and analyzed during the current study are available from the corresponding author on request.
